# Supplementary material for: PRSSLY-Based Molecular Sex Determination of Syrian Hamster (Mesocricetus auratus) Pups Using Placental Tissues
Source: Genes (Basel). 2026 Jan 28;17(2):143. doi: 10.3390/genes17020143 (PMC12940834; doi:10.3390/genes17020143)
Supplement: Supplementary file 1 [file genes-17-00143-s001.zip › Supplementary Figures.pdf]

SUPPLEMENTARY INFORMATION

PRSSLY-Based Molecular Sex Determination of Syrian Hamster (*Mesocricetus auratus*) Pups Using Placental Tissues

Yana Kumpanenko, Lindsey Piessens, Victor Neven, Kai Dallmeier, and Yeranddy A. Alpizar

Supplementary Figures

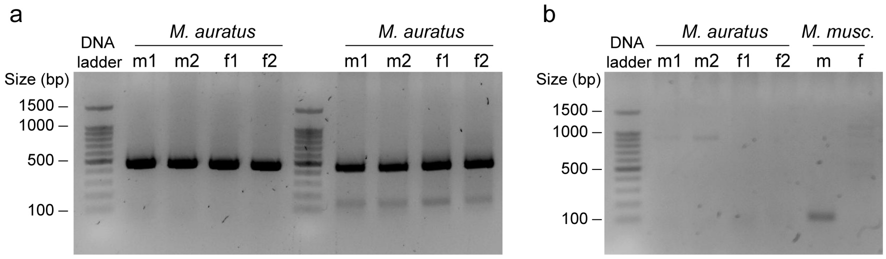

Supplementary Figure S1. Lack of sex-specific amplification in Syrian hamsters using universal *SRY/TDY* primers. **(a)** PCR amplification using two primer sets targeting the *SRY* gene. The SRYA-5 primer set is shown on the left side of the gel, and the SRY_Mm primer set on the right. Both reactions were performed using genomic DNA isolated from ear biopsies of adult Syrian hamsters. **(b)** PCR amplification using the maTdy primer set. Reactions were performed using genomic DNA isolated from ear biopsies of adult Syrian hamsters and adult mice. Across both panels, the universal primer sets did not produce sex-specific PCR products in Syrian hamsters. 100 bp DNA ladder; bp, base pairs.

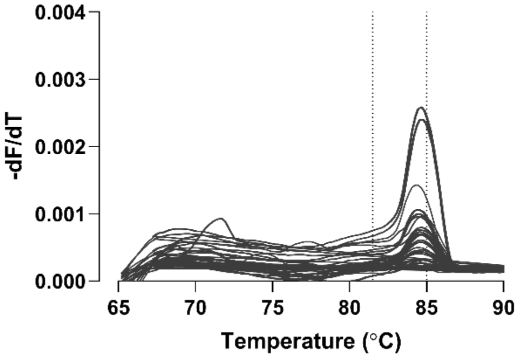

Supplementary Figure S2. Melt-curve analysis of placental gDNA using the Kdm5c primer set. Melt curves generated from placental gDNA using Kdm5c primers. Samples correspond to the same litter shown in Figure 4 A-C. Dotted vertical lines indicate the expected male-specific melt peak at 81.5 °C and the common peak at 85.0 °C, as defined in Meier *et al*. (2023).
